# The Effect of Alcohol and Sexual Arousal on Explicit and Implicit Condom Attitudes and Intentions to Use a Condom

**DOI:** 10.1007/s10508-022-02470-w

**Published:** 2022-11-28

**Authors:** Kenny Wolfs, Arjan E. R. Bos, Fraukje E. F. Mevissen, Jacques J. D. M. van Lankveld

**Affiliations:** 1 Faculty of Psychology, Open University, PO Box 2960, 6401 DL Heerlen, The Netherlands; 2 Faculty of Psychology and Neuroscience, Maastricht University, Maastricht, The Netherlands; 3 Municipal Public Health Service Rotterdam-Rijnmond, Department of Public Health, Rotterdam, The Netherlands

**Keywords:** Condomless sex, Alcohol, Sexual arousal, Working memory, Implicit attitudes

## Abstract

Alcohol and sexual arousal are contextual determinants of condomless sex. Dual-process theory postulates that two types of cognitive processing contribute to the regulation of behavior: one that is fast, intuitive and automatic, and another that is slower and deliberative. This study applied a dual-process model to investigate condomless sexual behavior, highlighting the potential importance of implicit attitudes in condomless sex. We investigated whether the impact of alcohol and sexual arousal on condom use-related attitudes and intentions was explained by diminished working memory capacity, as dual-process models suggest. We also investigated whether this effect could be explained by implicit and explicit attitudes toward condom use. Male participants (*N* = 30) were randomized using a 2 × 2 within-subjects design that manipulated alcohol intoxication (placebo vs. alcohol beverages) and sexual arousal (neutral vs. erotic movie clips). We measured participants’ working memory capacity, intentions to use a condom, and explicit and implicit attitudes toward condom use. Significant main effects of alcohol intoxication and sexual arousal on working memory capacity were found. No significant interaction was found for the combined effect of alcohol intoxication and sexual arousal on intentions to use a condom. There was no significant effect of implicit attitudes on intentions to use a condom, although a trend toward significance (*p* = 0.06) was found for the effect of implicit attitudes on intentions to use a condom when participants were in a state of alcohol intoxication. Theoretical and practical implications of this study are discussed.

## Introduction

Condomless sex, which leaves sexual partners unprotected against sexual transmission of infections and unwanted pregnancy, is still an important health-related hazard. Globally, large numbers of people attain sexually transmitted infections (STI’s) on a daily base. For example, the European Centre for Disease Prevention and Control (ECDC, 2014) estimated this number in Europe to be around 380,000 per year. The majority of sexually transmitted infections are attained by means of condomless sex (UNAIDS, 2016). Theoretical models and interventions to explain and reduce condomless sex have mostly been based on cognitive models of behavior, such as the theory of planned behavior (Ajzen, 1985). However, more and more evidence accumulates indicating that contextual factors also play an important part in determining condomless sex.

Determinants of condomless sex have been investigated in studies using global, situational, event-level, and experimental designs. Compared with global designs, situational and event-level studies enable investigating contextual determinants of condomless sex. However, these designs do not allow for causal inferences regarding the effects on condomless sex of the factors of interest, while experimental study designs do (Palfai & Luehring-Jones, 2021). We note that the literature is skewed toward global and situational designs. Event-level studies have linked alcohol consumption to number of occasions of unsafe sex (Cooper, 2002, 2006; Cooper & Orcutt, 1997; Davis et al., 2009; Leigh, 2002). The experimental literature on alcohol intoxication and intentions to use condoms is still scarce (for a review, see Scott-Sheldon et al., 2016). The available experimental evidence strongly suggests that alcohol lowers people’s intentions to use a condom (Davis et al., 2015; MacDonald et al., 2000; Maisto et al., 2012; Norris et al., 2009; Scott-Sheldon et al., 2016; Wray et al., 2015). In a series of experiments, George et al. (2009) showed that the effect of alcohol was fully mediated by sexual arousal. More specifically, alcohol consumption led to subjective feelings of sexual arousal, which in turn resulted in stronger intentions to engage in unsafe sex. This mediating effect of sexual arousal was replicated by (Davis et al., 2009). Sexual arousal moderated the associations of implicit and explicit condom use attitudes with condom use intentions in an experimental study among undergraduate students (Wolfs et al., 2019). When the participants were not aroused, their intentions to use a condom were exclusively predicted by their explicit attitudes toward condoms. However, in a sexually aroused state, condom use intentions were predicted by both explicit and implicit attitudes.

Alcohol use and sexual arousal should therefore both be taken into consideration when trying to understand why people exhibit unsafe sexual behavior. Although higher alcohol expectancies were found to predict stronger intentions for condomless sex (Fromme et al., 1999; Palfai & Luehring-Jones, 2021), the findings mainly came from cross-sectional studies. However, Scott-Sheldon et al. (2016), in their meta-analysis of the available experimental evidence from studies into the link between alcohol use and condom use intentions, concluded that alcohol use itself, but not alcohol expectancies, was causally related to greater intentions for condomless sex.

### The Reflective-Impulsive Model

To understand how contextual influences have an effect on condom use, a dynamic model of condomless sex is needed. Dual-process models seem to be especially suited to do so. An application of these models is the Reflective Impulsive Model (RIM: Strack & Deutsch, 2004). According to the RIM, behavior is a joint outcome of two parallel cognitive systems; the reflective and the impulsive system. The impulsive system is conceptualized as an associative network in which information is processed automatically and effortlessly. Associations in the impulsive system are strengthened when stimuli appear in close proximity of each other. The impulsive system appears like a simple memory system representing simple perceptual concepts. For example, after having had multiple pleasurable sexual encounters without a condom, sex-related cues would make one long for sex without a condom. An example of such cue could be the perception of genital sexual arousal. Sexual arousal would then be linked to the act of sex in the associative network, which would in turn trigger the impulsive behavior to have sex without a condom.

The reflective system, on the other hand, relies on logic and rule-based reasoning for processing information. It requires mental effort and can only operate when sufficient cognitive resources are available. It generates explicit judgments, attitudes, and decisions that, put together, form action plans in new situations (Lieberman et al., 2002; Strack & Deutsch, 2004). For example, given that the goal is to protect oneself from sexually transmitted infections, the reflective system has an influence on condomless sex based on logical reasons rather than associative cues. It can override impulses to have sex without a condom. To do so, the reflective system needs to be able to flexibly interpret concepts and link them together in new ways to form new meanings and behavioral outcomes (e.g., “Sex without a condom would be more pleasurable, but I don’t know this person’s STI status and I know STI’s are prevalent, and thus I should use a condom in this sexual encounter”) (Hummel & Holyoak, 2003). To be able to override the impulsive system, the reflective system requires mental effort. In the present study, in accordance with the RIM (Strack & Deutsch, 2004), mental effort is operationalized as working memory capacity. With a low working memory capacity, the reflective system can no longer override the impulsive system, and the latter will have a larger influence on the behavior at hand.

The negative effects of alcohol on working memory and executive functioning have been extensively documented. Alcohol negatively affects executive functioning in general, including cognitive processes such as selective attention, task switching, and working memory (Baddeley et al., 2001; Paulus et al., 2006; Saults et al., 2007; Schweizer et al., 2006; Weissenborn & Duka, 2003). Moderate doses of alcohol have been linked to decreases in visual as well as auditory working memory capacity (Saults et al., 2007). A dose–response association of executive function performance with increasing blood alcohol level (BAL) has been demonstrated (Finn et al., 1999). Although most studies showed a negative effect of alcohol on working memory capacity, mixed results have also been found. Schweizer et al. (2006) failed to find a significant effect of alcohol on a verbal task, but did identify its effect on a visual-spatial memory task. However, when using a different visual-spatial memory task, Paulus et al. (2006) found no effect of alcohol. Another study using a pattern-recognition task only found an effect of alcohol on respondent's planning ability, but not on their working memory (Weissenborn & Duka, 2003). An alternative explanation for these mixed results might be that trait working memory had a confounding effect. A study using the backward digit-span test found that the effect of alcohol performance was limited to individuals with a high trait working memory (Finn et al., 1999). In short, most scientific evidence indicates that alcohol intoxication reduces working memory capacity.

While several studies have been published on the effect of alcohol on working memory capacity, there are no studies that directly investigated the effect of sexual arousal on working memory capacity. However, indirect support for such effect has been found. van Lankveld and Smulders (2008) found that sexual pictures capture attention as shown by a lower P300-signal on an oddball task. This finding was corroborated by Carvalho et al. (2011) using movie clips instead of pictures. In their experiment, sexually arousing movie clips also caused a larger decrease in the P300-signal than neutral movie clips. We hypothesize that sexual arousal will thus have a negative effect on working memory capacity, because it captures attention. Consequently less working memory capacity remains available for explicit cognitions about safe sex.

In this study, we explored whether the Reflective-Impulsive Model can explain the negative effect of alcohol and sexual arousal on intentions to use a condom. We investigated a sample of male students, because we expected stronger effects in men, as compared with women (Scott-Sheldon et al., 2016). A first step is to check whether the assumption based on the RIM, that being in a state of alcohol intoxication or sexual arousal reduces working memory capacity. If alcohol and sexual arousal reduce working memory capacity, we still need to investigate whether working memory capacity has a moderating effect on the influence of both explicit and implicit cognitions on intentions to use a condom. The model predicts that, if participants are in a neutral state and have ample working memory capacity at their disposal, intentions to use a condom will be determined solely by explicit cognitions. If, however, working memory capacity is reduced, implicit cognitions regarding condom use will have a larger effect on intentions to use a condom. We tested the following hypotheses: (1) Alcohol and sexual arousal reduce working memory capacity. The effects of alcohol and sexual arousal on working memory capacity are additive. (2) Alcohol and sexual arousal have a negative effect on intentions to use a condom. (3) There is a main effect of explicit attitudes on intentions to use a condom. This main effect represents the model’s assumption that when people are not under the influence of alcohol or sexual arousal, their behavior will be predicted by their explicit cognitions. (4) Alcohol intoxication moderates the effect of implicit attitudes and explicit attitudes toward condom use on intentions to use a condom. This represents the model’s assumption that, when people are under the influence of alcohol and therefore have a diminished working memory capacity, implicit associations will have a larger effect on intentions to use a condom. Explicit attitudes will have a smaller effect on intentions to use a condom compared to when participants are in a neutral state. (5) There is a moderating effect of sexual arousal on the effect of implicit attitudes and explicit attitudes toward condom use on intentions to use a condom. This represents the model’s assumption that, when people are sexually aroused and therefore have a diminished working memory capacity, implicit associations will have an effect on intentions to use a condom. Explicit attitudes will have a smaller effect on intentions to use a condom compared to when participants are in a neutral state.

## Method

### Participants

Participants were recruited at Maastricht University using (digital) flyers as well as via several Facebook groups of Maastricht University students specifically set up to recruit participants for scientific studies in multiple disciplines. Participants received a monetary compensation of €75. Inclusion criteria were age 18 to 35, male and heterosexual (or at least being turned on by straight porn). In total, 30 participants were tested. All reported being heterosexual. Participants had a mean age of 23.50 (*SD* = 4.20). Most (*N* = 14) had a German background, four participants were Dutch, and ten participants were in the “other” category, which for example included people with a Brazilian, Czech and American background. Most participants were single (*N* = 18), ten participants were in a steady relationship while two participants reported being in an open relationship. The average lifetime number of sexual partners with whom they had unsafe sex at least once was 6.63 (*SD* = 10.30, median = 3). Table 1 presents an overview of the participants’ main baseline characteristics. Participants scored average on trait working memory, with the exception of the first task (repeating letters), which was performed slightly above average as defined by the standard scores of the WAIS-III (see Measures for more information). Participants had rather positive attitudes toward condoms, except for pleasure, which is the only attitude with a mean score that falls below half of the possible range on this subset of questions. To investigate whether relationship status was related to condom use intentions (e.g., Mevissen et al., 2011; Protogerou & Turner-Cobb, 2011), a binary logistic regression analysis was performed with relationship status (single or open relationship vs. steady relationship) as to-be-predicted variable. Relationship status was not associated with condom use intentions at baseline.

**Table 1:** Baseline measurements of explicit attitudes toward condoms, trait working memory, and demographic variables

Measure	Range	Min	Max	Mean	SD
Age	18–35	19	33	23.50	4.20
Number of partners with unsafe sex	0–50	0	50	6.63	10.30
*MCAS*					
Reliability	5–35	17	35	29.03	3.86
Pleasure	5–35	5	29	18.30	5.90
Stigma	5–35	5	35	30.27	5.66
Embarrassment (negotiation)	5–35	5	35	29.80	5.75
Embarrassment (purchase)	5–35	8	35	27.47	6.45
*Standardized WAIS scores*					
Total	3–60	6	42	33.83	4.73
Repeating numbers	1–20	17	17	12.23	2.85
Calculus	1–20	6	15	10.40	2.19
Letters and numbers	1–20	7	15	11.20	1.80

### Design

This study used a 2 × 2 within-subjects design with alcohol intoxication (placebo vs. alcohol beverages) and sexual arousal (neutral vs. erotic movie clips) as within-subjects factors. The order of the alcohol intoxication and sexual arousal conditions was randomized using a Latin square. The experiment consisted of two sessions, separated by a week, that were identical to each other, with the exception that the first session contained baseline measures of trait working memory capacity.

### Materials

### Experimental Manipulation

#### Alcohol Administration

After completing baseline measures, participants were weighed and were asked to consume three beverages, that either contained alcohol or placebo. The placebo drinks contained a mix of orange juice and flattened tonic water, following the protocol used by Norris et al. (2009) and George et al. (2009). The bitterness of the flattened tonic water mimics the taste of alcohol. Also, one ml of vodka was carefully placed on top of the drink, so that the first sip of the drink would taste like it contained vodka, to make it harder to distinguish the placebo drink from the alcoholic drink. Adding this small spoon of vodka did not increase the BAL, as all participants in this condition scored a BAL of 0.00 on the breathalyzer test after beverage consumption. In the alcoholic drinks, the flattened tonic water was replaced with vodka. We aimed at maintaining a blood alcohol level of 0.05 until the end of the experiment (two hours after consumption), because this conforms to the legal limit in traffic in the Netherlands, ethical approval could not be obtained for higher doses, and higher doses might interfere with genital sexual arousal (Cooper, 2002; George et al., 2009). Although higher alcohol dosages have been linked to stronger decreases in executive functioning (Finn et al., 1999), the chosen dose should be able to reduce (visual) working memory capacity (Saults et al., 2007). We used the Widmark equation that takes the consumer’s weight and gender into account to estimate the required volume of alcohol to reach a certain blood alcohol level. Drinks in this condition were prepared by adding the required amount of vodka in a glass of orange juice and flattened tonic water. Participants were asked to consume a total of three beverages and to finish each beverage within approximately five minutes.

#### Sexual Arousal

Sexual arousal was induced by having participants watch a 5-min erotic movie clip. The erotic movie clip showed a heterosexual couple engaging in oral sex and subsequently in (unprotected) vaginal penetration. The clips were pilot-tested for being sufficiently sexually arousing and have been used in other studies (e.g., Grauvogl et al., 2015; Wolfs et al., 2019). In the neutral condition, participants watched a 5-min clip from the television show BBC Earth showing documentaries on the arctic and on time-lapse photography.

To ensure participants in the experimental condition stayed aroused after watching the video clip, they heard the audio from an erotic movie via a headphone set while they performed the working memory task. In the control condition, participants heard the audio from the BBC Earth documentary. The n-back working memory task, which was used for measuring working memory capacity, is a demanding cognitive task and therefore will act as a distractor and lead to a decrease in sexual arousal (Geer & Fuhr, 1976). This decrease in sexual arousal is more apparent in genital sexual arousal compared to subjective sexual arousal (Abrahamson et al., 1985; van Lankveld & van den Hout, 2004).

### Measures

For the explicit measures (condom use intentions, subjective sexual arousal, subjective alcohol intoxication and explicit attitudes), 100-mm visual analog scales (VAS) were used with two opposite statements at both sides of the line (e.g., “I do not feel sexually aroused at all” and “I feel very sexually aroused”). Participants were asked to place a mark on the scale indicating where they felt they were on the spectrum between the two extreme statements.

### Manipulation Checks

#### Genital Arousal

Genital arousal was assessed using an indium/gallium-in-rubber penile gauge. Changes in penile circumference were recorded as DC signal changes. The gauges were disinfected before each use, using Cidex^®^ OPA (Johnson & Johnson), a high-level disinfectant for medical devices. They were soaked for five minutes and then air-dried. Participants applied the plethysmograph themselves; they were instructed to place the strain gauge two-thirds of the way down the shaft of the penis toward the base. The gauges were calibrated after each laboratory session using a calibration cone.

#### Subjective Arousal

Subjective arousal was measured using one item: “How sexually aroused do you feel at the moment?” with “Not aroused at all” and “Very aroused” at both ends of a VAS.

### Alcohol Intoxication

An AlcoTrue C^®^ breathalyzer was used to measure the amount of alcohol in participants’ breath. It is CE-certified and comes with disposable mouthpieces to guarantee hygienic testing.

#### Subjective Intoxication

Subjective intoxication was measured using one item: “How intoxicated do you feel at the moment?” with “Not at all intoxicated” versus “Very intoxicated” at both sides of a VAS. After participants had consumed three beverages, they estimated how many of those beverages contained alcohol (0, 1, 2, or 3 glasses) using a forced choice question “How many of these beverages do you think contained alcohol?”

### Working Memory

#### Trait Working Memory

Trait working memory was assessed using three tasks that belong to the working memory capacity subset of the WAIS-III. The WAIS-III in general, as well as the subset of working memory tasks, has been validated (Tulsky & Price, 2003). The first task required the participants to remember a sequence of letters that they had to repeat either in forward or backward order. The second task consisted of several math problems that were read out aloud and that participants had to solve without taking any notes. During the final task, participants first listened to a sequence of characters and numbers, then repeated the sequence of characters first in chronological order, and then in alphabetical order. Using the manual of the WAIS-III (Wechsler, 1997), scores were standardized according to the participants’ age group.

#### State Working Memory

State working memory was measured using an n-back task (Jaeggi et al., 2003). Inquisit 3 was used to run the n-back working memory task. During this task, participants had to remember if the visual stimulus, a character they saw on screen was the same as they saw N stimuli ago. The value of N was two, three, and four in three consecutive blocks of trials. Before the test trials started, participants practiced the task for all three values of N. After this practice, they received feedback on how well they performed. Once the actual task started, feedback was no longer provided. The script of Jaeggi et al. (2003) immediately calculated the score on the working memory task, by subtracting the number of missed trials from the number of correct trials. The outcome of this difference was then divided by 5. Higher scores represent larger state working memory.

### Outcome Variables

#### Implicit Associations

Implicit condom attitudes were assessed using an Implicit Association Test (Greenwald et al., 2003) consisting of 5 blocks. In the first block (18 trials), participants categorized words in two attribute categories (positive/negative) that were displayed in the upper left and right corners of the computer screen. The positive category was represented by the words “Gift,” “Peace” and “Healthy,” while the negative category was represented by the words “Hate,” “War” and “Disease.” In the second block (congruent practice, 36 trials), participants categorized words into the same attribute categories, while a second target category of pictures was also introduced. Target labels were “safe sex” (penetration with a condom) and “unsafe sex” (penetration without a condom). These categories were represented by three pictures each, displaying either a penetration with or without a condom. The setup of the third block (congruent test, 48 trials) was similar to the second block. In the fourth block (incongruent practice, 36 trials), the positions of the attribute categories (e.g., safe sex/unsafe sex) on top of the screen were swapped. In order to categorize positive/negative words, participants now had to press the opposite keys of the ones in blocks one to three. The positions of “safe sex” and “unsafe sex” remained the same. The fifth (incongruent test) block (48 trials) had the same setup as the fourth block.

Calculation of Guttman’s split-half reliability was performed after removing latency scores below 400 ms and above 2500 ms. Coefficients for the IAT test trials (blocks 3 and 5) were 0.92 and 0.97 in the neutral condition and 0.95 and 0.96 in the erotic condition. These findings indicated satisfactory reliability (Bluemke & Friese, 2008).

#### Explicit Attitudes

Participants were asked to imagine they met someone at a local bar with whom they really hit it off. After talking for a while, they then proceeded to head home, and an opportunity for sex arose. We then asked participants to rate on a VAS how wise (very unwise – very wise), pleasant (very unpleasant – very pleasant), hard (very hard – very easy) or good (very bad idea – very good idea) it would be to use a condom in this situation. VAS scores on all items were summed up. The internal consistency for this scale in the current sample was high (α = 0.84).

#### Condom Use Intentions

Intentions were also measured using a VAS. To reduce the potential impact of participants’ relationship status on the measurement of condom use intentions participants were primed with a script depicting a casual sexual encounter. Our condom use intention scale consisted of three items. Participants first answered a question assessing how likely it would be that they would use a condom in the future (very unlikely–very likely), unrelated to the aforementioned imaginary situation. Then, they were asked whether they intended to use a condom in the aforementioned imaginary situation (very unlikely–very likely). Next question was how much trust they had that they would use a condom, even if their casual partner would not like to in that situation (very little trust–a lot of trust). Finally, we asked them whether, if there was no condom present, and there was no way of obtaining one, they would still have sex in this situation (very unlikely–very likely). The internal consistency for this scale was insufficient (*α* = 0.33). However, after deleting the first item (I intend to use a condom in the future), *α* was found to rise to 0.79. The first item seems to measure general intentions to use a condom, whereas the other three items are situational intentions to use a condom. We therefore deleted the first item and retained three situational items to measure intentions to use a condom. We also investigated the external validity of this scale by correlating the scale scores with past behavior, quantified as the number of sexual partners they had had unsafe sex with. For this purpose participants answered the question “How many different partners have you had unsafe sex with in the past?” A significant negative correlation was found between number of unsafe partners and intentions to use a condom (*r* = − 0.49; *p* = 0.007), suggesting satisfactory convergent validity of the scale.

#### Risk Perception

Risk perception was measured using a two-item scale. We asked participants to estimate the chance that they would ever attain an STI in their life (very small–very large chance). Using the same situation that was used to assess the explicit attitudes, we asked them how large they believed the chance was their partner would have an STI and how large the chance was they would attain an STI from this encounter. The internal consistency of scale with these three items was low (α = 0.618), but after deleting the first item (“How likely is it that you will get an STI at one point in your life?”) the internal consistency rose to α = 0.85. We therefore deleted the general item from the scale and retained the two situation-specific items.

### Procedure

Upon entering the laboratory, participants were asked to read an information letter, explaining that the study was intended to measure the effect of sexual arousal and alcohol on working memory, reaction times to computer tests and condomless sex. Then, they were asked to sign an informed consent statement. Subsequently, it was checked whether they were sober using the AlcoTrue C^®^ breathalyzer (Bluepoint-Medical, Selmsdorf Germany).

Three drinks were them consumed. One minute after participants finished their third drink another breathalyzer test was administered, to assure there was no lingering alcohol present in participants’ saliva that could confound the measurement. Next, they were verbally instructed how to apply the penile plethysmograph, then privately applied the plethysmograph. Next, a baseline penile circumference measurement was performed. Subsequently, participants rated their level of subjective sexual arousal, their subjective level of intoxication, and how many of the drinks they had consumed they believed contained alcohol.

After all baseline measures were completed, participants viewed a five-minute video clip that was either erotic or neutral in nature. They wore headphones to hear the sound of the movie clip while watching the video on screen. After they watched this video, without any further instruction participants were asked to complete measures of explicit attitudes, risk perception, and intentions. Next, the n-back task was administered on screen while participants still could hear the audio from the video clip through the headphones but were not shown the visual content. This was done in order to try and maintain sexual arousal during the working memory task (Geer & Fuhr, 1976). Finally, participants performed an Implicit Association Test to assess their implicit attitudes toward condom use.

After participants had completed one condition (erotic or neutral), they were given a break to wash out any carry-over effects from the sexual arousal manipulation. During this ten-minute break, they were asked to play some casual games on a tablet computer. The sexual arousal washout period has been used before in within-subjects designs of studies into sexual arousal effects on condom use intentions (e.g., Wolfs et al., 2019). After this break, participants completed the other condition. This concluded the first day of testing for participants.

Participants were asked to return to the laboratory for a second session exactly one week later (same day of the week and same hour). This second session was identical to the first one with the exception that there were no baseline measurements at the beginning of the session and that participants were assigned to the opposite beverage conditions in the second session. The order of beverage condition and sexual arousal condition was counterbalanced between participants.

This protocol was approved by the medical ethical committee of the Zuyderland Hospital in Heerlen, the Netherlands, and by the Ethics Review Committee Psychology and Neuroscience at Maastricht University, the Netherlands.

### Statistical Analysis

We used IBM SPSS 24 for all statistical analyses. Firstly, average explicit attitude scores were calculated. VAS scores were reversed if necessary to ascertain that higher scores represented a positive attitude toward condom use or a strong intention to use a condom. Average intentions to use a condom and risk perception were also measured using a VAS and their average score was calculated the same way. The D600-score was calculated for the reaction times on the Implicit Association Test, according to the formula of (Greenwald et al., 2003). Reaction times (RTs) below 400 ms, that are considered to reflect outliers due to random responding, were discarded and RTs higher than 2500 ms, presumedly reflecting responses during slips of attention, were replaced with 2500 ms. Error trial RTs were replaced with the mean RT of the participant’s correct responses in the same block in which the error occurred plus a 600 ms penalty. Positive D600-scores of the test trials represent a positive implicit attitude toward condom use.

For regression analyses, an unstructured covariance matrix was used, with a random intercept and a random slope per person. Predictor variables were centered before they were entered into the regression models. To test the first hypothesis, the score on the n-back task was used as dependent measure, sexual arousal and alcohol were entered as fixed factors, while trait working memory was inserted into the model as a covariate.

To test the second hypothesis, a mixed regression analysis was used. An unstructured covariance matrix was used, with a random intercept and a random slope per person. Intentions to use a condom was used as a dependent variable, and sexual arousal and alcohol were used as fixed factors.

Hypotheses three, four and five were tested using one single mixed regression analysis. An unstructured covariance matrix was used, with random intercept, and a random slope per person. Explicit attitudes, implicit attitudes, risk perception, and the interactions between implicit attitudes and condition, and between explicit attitudes and condition were used as covariates.

#### Statistical Power Calculation

Assuming a medium-size effect (d = 0.25) and a medium-size correlation between measurements of 0.5, a power of β = 0.80 was achieved for the two-way within-subjects interaction term with a minimum sample size of 28 participants. Condom use intentions were measured four times during each experimental condition. Correlation coefficients of condom use intention scores within each condition ranged from 0.20 ≤ *r* ≤ 0.78 (average *r* = 0.43), confirming the estimation used in our power analysis.

## Results

### Manipulation Check

In the neutral condition, changes in penile circumference, measured in mm, ranged from − 4.74 to 192.08. Since this increase of 192 mm in the neutral condition is an extreme outlier and is most likely due to a measurement error rather than an actual increase, data of this participant were omitted in further data processing. Of the remaining participants, changes in penile circumference in the neutral condition ranged from − 4.74 to 14.38 (M = 1.52; SD = 3.92). In the erotic condition, changes in penile circumference ranged from − 2.04 to 21.73 (M = 4.96; SD = 4.94).

In the sober condition, BAL was 0 for all participants. However, subjective intoxication level in this condition ranged from 0 to 66 (M = 20.98; SD = 19.83). In the alcohol condition, BAL ranged from 0.20 to 0.64 (M = 0.49; SD = 0.11). Subjective intoxication level ranged from 9.00 to 100.00 (M = 55 > 82; SD = 24.33) in the alcohol condition.

Significant differences were found between conditions for BAL (*t*(28) = 23.7; *p* < 0.001), subjective intoxication (*t*(27) = 7.28; *p* < 0.001), genital arousal (*t*(27) = 5.49; *p* < 0.001), and for subjective arousal (*t*(28) = 4.87; *p* < 0.001).

### The Effects of Alcohol and Sexual Arousal on Working Memory Capacity

**Fig. 1:**
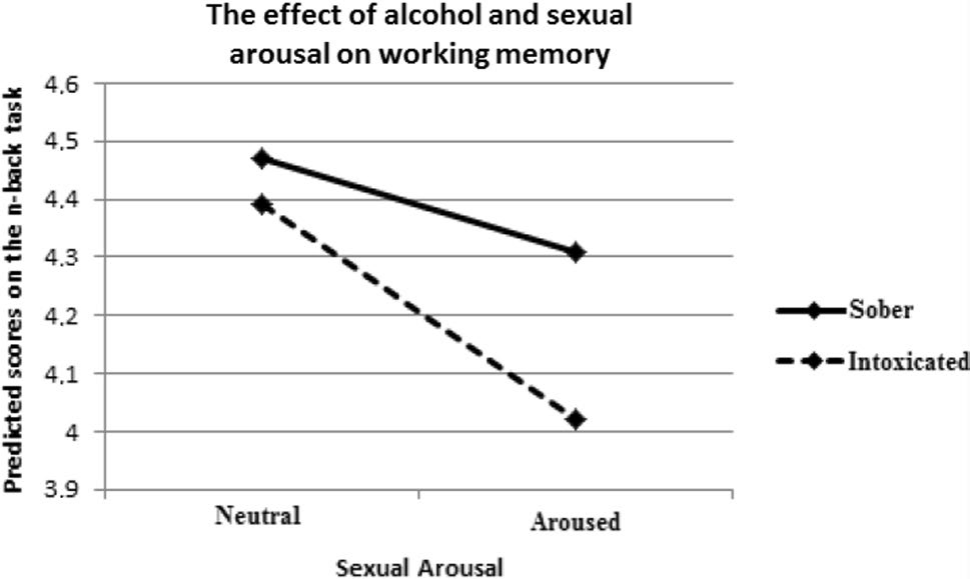
Graphic representation of the effect of alcohol and sexual arousal on working memory

Descriptive statistics and bivariate correlation coefficients of the key variables are shown in Table 2. Explicit condom attitudes were found to have the strongest associations with condom use intentions across conditions (0.79 ≤ *r* ≤ 0.77). The association of implicit condom attitude with condom use intentions was only significant in the condition with placebo drinks and neutral video (*r* = 0.41, *p* < 0.05). Main effects on state working memory capacity were found of alcohol intoxication (*t*(28) = 3.40; *p* = 0.01), and of sexual arousal (*t*(28) = 2.32; *p* < 0.05). State working memory capacity was found to be lower at higher levels of alcohol intoxication and sexual arousal. We also found a significant interaction effect of alcohol and sexual arousal on state working memory capacity (*t*(28) = 2.83; *p* = 0.01). The details of this analysis can be found in Table 3 with the alcohol + sexual arousal condition as referencing condition and are visually shown in Fig. 1. All differences that can be seen in Fig. 1 are significant.

**Table 2:** Descriptive statistics and bivariate correlations of key variables across experimental conditions

	M	SD	Correlations			
*Condition: Placebo + Neutral*			1	2	3	4
1 Condom Use Intention	58.18	25.72	–			
2 Implicit Condom Attitude	0.10	0.34	0.41*	–		
3 Explicit Condom Attitude	71.94	17.48	0.77***	0.44*	–	
4 State Working Memory	4.17	0.50	0.07	− 0.21	0.16	–
*Condition: Placebo + Arousal*						
1 Condom Use Intention	56.02	27.16	–			
2 Implicit Condom Attitude	0.21	0.34	0.02	–		
3 Explicit Condom Attitude	71.13	18.13	0.77***	0.14	–	
4 State Working Memory	4.03	0.62	0.17	0.19	0.19	–
*Condition: Alcohol + Neutral*						
1 Condom Use Intention	55.63	26.03	–			
2 Implicit Condom Attitude	0.34	0.33	0.20	–		
3 Explicit Condom Attitude	72.16	18.53	0.70***	0.29	–	
4 State Working Memory	4.11	0.73	− 0.16	− 0.05	0.04	–
*Condition: Placebo + Arousal *						
1 Condom Use Intention	54.08	26.12	–			
2 Implicit Condom Attitude	0.24	0.29	0.25	–		
3 Explicit Condom Attitude	69.94	20.24	0.71***	0.39*	–	
4 State Working Memory	3.71	0.83	− 0.11	0.27	0.09	–

*p* ≤ 0.05; ** = *p* ≤ 0.01; *** = *p* ≤ 0.001

**Table 3:** The results of mixed regression analysis with scores on the n-back task as dependent variable

Parameter	Estimate	Standard error	*t*	*p*
Intercept	4.47	0.62	6.45	< 0.001
Placebo–Neutral^a^	0.45	0.16	2.83	0.008
Placebo–Erotic^a^	0.31	0.14	2.32	0.028
Alcohol–Neutral^a^	0.39	0.12	3.40	0.002
Baseline working memory	− 0.01	0.02	− 0.52	0.610

^a^Tested against Alcohol-Erotic as reference condition

### The Effects of Alcohol and Sexual Arousal on Intentions to Use a Condom

We found no significant effect of sexual arousal on intentions to use a condom, but a significant effect was found of alcohol (*t*(28) = 2.55; *p* = 0.01. As expected, intentions to use a condom were lower under the influence of alcohol. Full results of this analysis can be found in Table 4.

**Table 4:** Results of mixed regression analysis with intentions to use a condom as dependent variable

Parameter	Estimate	Standard error	*t*	*p*
Intercept	− 20.34	32.13	− .633	1.000
Alcohol	12.688	4.97	2.549	0.012
Sexual arousal	0.003	4.77	0.001	0.999
Alcohol * Sexual arousal	− 1.98	3.71	− .53	0.597
Explicit attitudes	0.478	0.11	4.556	< .001
Implicit attitudes	0.322	0.11	0.092	0.927
Explicit * Alcohol	− 0.182	0.07	− 2.554	0.012
Explicit * Sexual arousal	− 0.020	0.07	20.197	0.773
Implicit * Alcohol	5.19	4.12	1.259	0.120
Implicit * Sexual arousal	0.096	3.79	0.025	0.980
Baseline working memory	1.28	0.814	1.573	0.118

### The Effects of Explicit and Implicit Attitudes on Intention to Use a Condom

Explicit attitudes were found to have a strong positive effect on intentions to use a condom (*t*(28) = 4.56; *p* < 0.01) when participants were sober and not sexually aroused, i.c. when their working memory capacity was undisturbed. There was no main effect of implicit attitudes on intentions to use a condom. No significant interaction effects were found, except for the interaction between alcohol and explicit attitudes toward condoms (*t*(28) = − 2.55; *p* = 0.01). This implies that when people are under the influence of alcohol, explicit attitudes have a smaller effect on intentions to use a condom compared to when they were sober. No significant interaction effect of implicit attitudes and any of the conditions was found. The results of the mixed regression analysis can be found in Table 4.

## Discussion

In this study, we tested a dual-process model of condomless sex to explain the negative effects of alcohol and sexual arousal on intentions to use a condom (George et al., 2009). Firstly, as hypothesized, working memory capacity appears to diminish when participants were under the influence of alcohol and when they were sexually aroused. They performed worse on an n-back working memory task (Jaeggi et al., 2003), compared to the neutral condition. When participants were intoxicated as well as sexually aroused, they performed even worse than when they were only intoxicated or sexually aroused. These findings are in line with earlier research on the effect of alcohol on working memory capacity (e.g., Baddeley et al., 2001; Weissenborn & Duka, 2003). This study is, to our knowledge, the first to directly test the effect of sexual arousal on working memory capacity. We want to be cautious, however, in claiming that the working memory capacity that is required for adequate decision making in the heat of the moment is impaired by intoxication and sexual arousal, due to the laboratory context of the findings. The results align with previous findings that sexual arousal captures attention (Carvalho et al., 2011; van Lankveld & Smulders, 2008). A limitation was that an erotic audio stimulus was delivered to maintain the level of sexual arousal while participants performed the working memory task, which might have distracted participants from achieving their normal level of performance on the task, resulting in less valid measurement of working memory capacity. In summary, the first assumption of the dual process model to explain the effect sexual arousal and alcohol have on condomless sex received support.

The effect of a state of alcohol intoxication on condom use intentions was found significant, underscoring findings in previous research (Davis et al., 2015; MacDonald et al., 2000; Maisto et al., 2012; Norris et al., 2009; Scott-Sheldon et al., 2016; Wray et al., 2015). However, contrary to our expectations and suggestions based on prior research (Davis et al., 2009; George et al., 2009), induction of a state of sexual arousal was not found to impact condom use intentions in this study. The failure to find the expected effect cannot readily be explained by ineffectiveness of the sexual arousal induction method, given the observed effects in this condition on working memory capacity and on subjective and genital sexual arousal. An explanation might be that the effect of sexual arousal on condom use intentions is substantially smaller than that of alcohol intoxication and that the study was not sufficiently powered to identify this smaller effect.

In addition to the effects of alcohol and sexual arousal on working memory capacity, and in accordance with a dual-process model of condomless sex, we found a large impact of explicit condom attitudes on intentions to use a condom when participants were in a neutral state; the effect of explicit attitudes on intentions to use a condom diminished when participants were intoxicated. We did not find evidence that implicit condom attitudes had a direct effect on intentions to use a condom, nor when participants were under the influence of alcohol or sexually aroused. These findings did not replicate earlier findings that implicit attitudes affected intentions to use a condom (Ajzen et al., 2007; Wolfs et al., 2019). According to the dual-process model, the effect of implicit attitudes on condom use should be maximal when working memory capacity is fully occupied. An obvious explanation for not finding this effect is that the study was underpowered. There is little information on the size of the effects of implicit attitudes on condomless sex, and the estimated effect size used in our a priori power calculation might have overestimated the effect.

However, assuming our test had enough power, another explanation may be that participants had to complete visual analogue scales four times during the experiment, twice per test day, potentially causing a memory bias, when they wanted to remain consistent in their answers.

This raises the question whether our manipulations were successful in reducing working memory capacity. In the condition where participants were both intoxicated and sexually aroused, participants on average scored higher than 4 on a scale ranging from 0 to 5. It should be noted that we deliberately chose the easy version of the n-back working memory test by Jaeggi et al. (2003), so it would not interfere with sexual arousal, which could also explain the higher level of subjective sexual arousal. Alternatively, the study dose of alcohol perhaps did not sufficiently reduce working memory capacity. We chose the administered dose of alcohol to raise the BAL up to the legal limit in traffic in the Netherlands, which should be able to reduce (visual) working memory capacity, but higher alcohol dosages have been linked to stronger decreases in executive functioning (Finn et al., 1999). Finally, one could question whether a BAL of 0.05 can be considered to be ecologically valid in a student population.

In this study, explicit attitudes toward condom use were a significant predictor of intentions to use a condom in all four experimental conditions. This finding supports the predictions based on the Theory of Planned Behavior and the Reasoned Action Approach, although it does highlight the importance of taking contextual factors, such as alcohol, into account when using the Theory of Planned Behavior or Reasoned Action Approach to explain condom use. Considering that alcohol use did weaken the effect of explicit attitudes on intentions to use a condom, a dual-process model of condom use is a promising and useful theoretical framework to explain condomless sex in different situations. According to this model of condom use, however, we would also expect an interaction effect between implicit attitudes and alcohol. We did not find this interaction effect in this study, but this does not mean we should easily reject a dual-process account of condomless sex. An important limitation of this study is that intentions for condom use were investigated instead of actual behavior. Even though intentions are strongly linked to behavior, there is also an intention-behavior gap when it comes to condom use (Sniehotta et al., 2014). Other experimental studies of dual-process models that turned out to be conclusive have used actual behavior such as eating (Hofmann & Friese, 2008), and alcohol use (Thush et al., 2008) for example. There are of course ethical and practical reasons as to why we cannot measure condomless sexual behavior in the laboratory, but future research into dual-process models of condomless sex could apply a similar outcome measure for condom use as was used in the study by Thush et al. (2008). One of the outcome measures was drinking behavior in the following month after the experiment, which does not require the targeted behavior to be performed in the laboratory.

In conclusion, we aimed to test a dual-process model of condomless sex. We found support for key assumptions in the model, namely that alcohol intoxication and sexual arousal reduce working memory capacity, and that the effects of explicit attitudes on intentions to use a condom weaken when people consume alcohol. We found no evidence that implicit attitudes play a role in condomless sex. However, we do believe it is too soon to reject a dual-process model of condomless sex altogether. Implicit attitudes might still be an important focus in future studies into condomless sex. Dual-process models seem to be promising models to explain the contextual influences of alcohol (Davis et al., 2009, 2015) or sexual arousal (George et al., 2009; Norris et al., 2009) on condom use intentions, and should at least be the subject of future studies.

## Data Availability

Open Science Framework: https://osf.io/h6um5/?view_only=854a2feb41944286b97a0ca4db91e1e5

## References

[CR2] Abrahamson DJ, Barlow DH, Sakheim DK, Beck JG, Athanasiou R (1985). Effects of distraction on sexual responding in functional and dysfunctional men. Behavior Therapy.

[CR3] Ajzen I (1985). From intentions to actions: A theory of planned behavior.

[CR4] Ajzen I, Albarracin D, Hornik R (2007). Prediction and change of health behavior: Applying the reasoned action approach.

[CR5] Baddeley A, Chincotta D, Adlam A (2001). Working memory and the control of action: Evidence from task switching. Journal of Experimental Psychology: General.

[CR6] Bluemke M, Friese M (2008). Reliability and validity of the Single-Target IAT (ST-IAT): Assessing automatic affect towards multiple attitude objects. European Journal of Social Psychology.

[CR7] Carvalho S, Leite J, Galdo-Álvarez S, Gonçalves ÓF (2011). Psychophysiological correlates of sexually and non-sexually motivated attention to film clips in a workload task. PLoS ONE.

[CR8] Cooper ML (2002). Alcohol use and risky sexual behavior among college students and youth: Evaluating the evidence. Journal of Studies on Alcohol, Suppl.

[CR9] Cooper ML (2006). Does drinking promote risky sexual behavior?: A complex answer to a simple question. Current Directions in Psychological Science.

[CR10] Cooper ML, Orcutt HK (1997). Drinking and sexual experience on first dates among adolescents. Journal of Abnormal Psychology.

[CR11] Davis KC, Danube CL, Neilson EC, Stappenbeck CA, Norris J, George WH, Kajumulo KF (2015). Distal and proximal influences on men’s intentions to resist condoms: Alcohol, sexual aggression history, impulsivity, and social-cognitive factors. AIDS and Behavior.

[CR12] Davis KC, George WH, Norris J, Schacht RL, Stoner SA, Hendershot CS, Kajumulo KF (2009). Effects of alcohol and blood alcohol concentration limb on sexual risk-taking intentions. Journal of Studies on Alcohol and Drugs.

[CR45] European Centre for Disease Prevention and Control (ECDC). (2014). WHO Regional Office for Europe. *HIV/AIDS Surveillance in Europe 2013*. Stockholm, Sweden: European Centre for Disease Prevention and Control. www.ecdc.europa.eu

[CR13] Finn PR, Justus A, Mazas C, Steinmetz JE (1999). Working memory, executive processes and the effects of alcohol on Go/No-Go learning: Testing a model of behavioral regulation and impulsivity. Psychopharmacology.

[CR14] Fromme K, D'Amico EJ, Katz EC (1999). Intoxicated sexual risk taking: An expectancy or cognitive impairment explanation?. Journal of Studies on Alcohol.

[CR15] Geer JH, Fuhr R (1976). Cognitive factors in sexual arousal: The role of distraction. Journal of Consulting and Clinical Psychology.

[CR16] George WH, Davis KC, Norris J, Heiman JR, Stoner SA, Schacht RL, Hendershot CS, Kajumulo KF (2009). Indirect effects of acute alcohol intoxication on sexual risk-taking: The roles of subjective and physiological sexual arousal. Archives of Sexual Behavior.

[CR17] Grauvogl A, de Jong P, Peters M, Evers S, van Overveld M, van Lankveld J (2015). Disgust and sexual arousal in young adult men and women. Archives of Sexual Behavior.

[CR18] Greenwald AG, Nosek BA, Banaji MR (2003). Understanding and using the Implicit Association Test: I An improved scoring algorithm. Journal of Personality and Social Psychology.

[CR19] Hofmann W, Friese M (2008). Impulses got the better of me: Alcohol moderates the influence of implicit attitudes toward food cues on eating behavior. Journal of Abnormal Psychology.

[CR20] Hummel JE, Holyoak KJ (2003). A symbolic-connectionist theory of relational inference and generalization. Psychological Review.

[CR21] Jaeggi SM, Seewer R, Nirkko AC, Eckstein D, Schroth G, Groner R, Gutbrod K (2003). Does excessive memory load attenuate activation in the prefrontal cortex? Load-dependent processing in single and dual tasks: Functional magnetic resonance imaging study. NeuroImage.

[CR22] Leigh BC (2002). Alcohol and condom use: A meta-analysis of event-level studies. Sexually Transmitted Diseases.

[CR23] Lieberman MD, Gaunt R, Gilbert DT, Trope Y (2002). Reflexion and reflection: A social cognitive neuroscience approach to attributional inference. Advances in Experimental Social Psychology.

[CR24] MacDonald TK, Fong GT, Zanna MP, Martineau AM (2000). Alcohol myopia and condom use: Can alcohol intoxication be associated with more prudent behavior?. Journal of Personality and Social Psychology.

[CR25] Maisto SA, Palfai T, Vanable PA, Heath J, Woolf-King SE (2012). The effects of alcohol and sexual arousal on determinants of sexual risk in men who have sex with men. Archives of Sexual Behavior.

[CR26] Mevissen FEF, Ruiter RAC, Meertens RM, Zimbile F, Schaalma HP (2011). Justify your love: Testing an online STI-risk communication intervention designed to promote condom use and STI-testing. Psychology & Health.

[CR27] Norris J, Stoner SA, Hessler DM, Zawacki T, Davis KC, George WH, Morrison DM, Parkhill MR, Abdallah DA (2009). Influences of sexual sensation seeking, alcohol consumption, and sexual arousal on women's behavioral intentions related to having unprotected sex. Psychology of Addictive Behaviors.

[CR28] Palfai TP, Luehring-Jones P (2021). How alcohol influences mechanisms of sexual risk behavior change: Contributions of alcohol challenge research to the development of hiv prevention interventions. AIDS and Behavior.

[CR29] Paulus MP, Tapert SF, Pulido C, Schuckit MA (2006). Alcohol attenuates load-related activation during a working memory task: relation to level of response to alcohol. Alcoholism: Clinical and Experimental Research.

[CR30] Protogerou C, Turner-Cobb J (2011). Predictors of non-condom use intentions by university students in Britain and Greece: The impact of attitudes, time perspective, relationship status, and habit. Journal of Child & Adolescent Mental Health.

[CR31] Saults JS, Cowan N, Sher KJ, Moreno MV (2007). Differential effects of alcohol on working memory: Distinguishing multiple processes. Experimental and Clinical Psychopharmacology.

[CR32] Schweizer TA, Vogel-Sprott M, Danckert J, Roy EA, Skakum A, Broderick CE (2006). Neuropsychological profile of acute alcohol intoxication during ascending and descending blood alcohol concentrations. Neuropsychopharmacology.

[CR33] Scott-Sheldon LA, Carey KB, Cunningham K, Johnson BT, Carey MP (2016). Alcohol use predicts sexual decision-making: A systematic review and meta-analysis of the experimental literature. AIDS and Behavior.

[CR34] Sniehotta FF, Presseau J, Araújo-Soares V (2014). Time to retire the theory of planned behaviour [Editorial]. Health Psychology Review.

[CR35] Strack F, Deutsch R (2004). Reflective and impulsive determinants of social behavior. Personality and Social Psychology Review.

[CR36] Thush C, Wiers RW, Ames SL, Grenard JL, Sussman S, Stacy AW (2008). Interactions between implicit and explicit cognition and working memory capacity in the prediction of alcohol use in at-risk adolescents. Drug and Alcohol Dependence.

[CR37] Tulsky DS, Price LR (2003). The joint WAIS-III and WMS-III factor structure: Development and cross-validation of a six-factor model of cognitive functioning. Psychological Assessment.

[CR38] UNAIDS. (2016). *Global AIDS Update 2016*. http://www.unaids.org/en/resources/documents/2016/Global-AIDS-update-2016

[CR39] van Lankveld J, Smulders FT (2008). The effect of visual sexual content on the event-related potential. Biological Psychology.

[CR40] van Lankveld J, van den Hout MA (2004). Increasing neutral distraction inhibits genital but not subjective sexual arousal of sexually functional and dysfunctional men. Archives of Sexual Behavior.

[CR41] Wechsler D (1997). Wechsler Adult Intelligence Scale—Third Edition (WMS-III).

[CR42] Weissenborn R, Duka T (2003). Acute alcohol effects on cognitive function in social drinkers: Their relationship to drinking habits. Psychopharmacology.

[CR43] Wolfs K, Bos AER, Mevissen FEF, Peters G-JY, van Lankveld JJDM (2019). Sexual arousal and implicit and explicit determinants of condom use intentions. Archives of Sexual Behavior.

[CR44] Wray TB, Simons JS, Maisto SA (2015). Effects of alcohol intoxication and autonomic arousal on delay discounting and risky sex in young adult heterosexual men. Addictive Behaviors.

