# Correction to: The Effect of Alcohol and Sexual Arousal on Explicit and Implicit Condom Attitudes and Intentions to Use a Condom

**DOI:** 10.1007/s10508-022-02511-4

**Published:** 2023-01-11

**Authors:** Kenny Wolfs, Arjan E. R. Bos, Fraukje E. F. Mevissen, Jacques J. D. M. van Lankveld

**Affiliations:** 1 Faculty of Psychology, Open University, PO Box 2960, 6401 DL Heerlen, The Netherlands; 2 Faculty of Psychology and Neuroscience, Maastricht University, Maastricht, The Netherlands; 3 Department of Public Health, Municipal Public Health Service Rotterdam-Rijnmond, Rotterdam, The Netherlands

**Correction to: Archives of Sexual Behavior** 10.1007/s10508-022-02470-w

Table 2 was missing from this article as originally published, while the table labeled “Table 2” was in fact Table 3.

The original article has been corrected.

